# Hypothesis-driven salvage therapy with corticosteroids, tofacitinib, and nintedanib for progressive radiation pneumonitis with possible immune-mediated contribution: a case report

**DOI:** 10.3389/fimmu.2026.1913167

**Published:** 2026-08-12

**Authors:** Xi Yu, Bowen Xu, Jiaxing Sun, Hongmei Wang, Yongzhong Guo, Dunqiang Ren

**Affiliations:** 1 Department of Respiratory and Critical Care Medicine, The Affiliated Hospital of Qingdao University, Qingdao, China; 2 Department of Respiratory and Critical Care Medicine, Xuzhou Central Hospital, Southeast University Affiliated Xuzhou Central Hospital, The Xuzhou School of Clinical Medicine of Nanjing Medical University, Xuzhou Clinical School of Xuzhou Medical University, Xuzhou, Jiangsu, China

**Keywords:** case report, immune checkpoint inhibitor, JAK inhibitor, nintedanib, radiation pneumonitis

## Abstract

Sequential or concurrent thoracic radiotherapy and immune checkpoint inhibitors (ICIs) increase the risk of severe overlapping pulmonary toxicity. Progressive pneumonitis after thoracic radiotherapy and ICI exposure remains a clinically challenging condition, particularly when it shows an inadequate response to systemic corticosteroid therapy. We report a 65-year-old man with esophageal cancer who developed progressive radiation pneumonitis with possible immune-mediated contribution after pembrolizumab therapy and thoracic radiotherapy. Despite moderate-dose systemic corticosteroid therapy, he experienced rapid clinical and radiographic deterioration. Laboratory evaluation showed a hyper-inflammatory phenotype, with elevated interleukin-6, ferritin, and C-reactive protein levels. Based on this hyper-inflammatory profile, we initiated a hypothesis-driven salvage regimen comprising corticosteroid therapy, the JAK inhibitor tofacitinib, and nintedanib. This intervention was associated with symptomatic improvement, declining inflammatory biomarkers, and near-complete radiographic resolution. No clinically evident severe treatment-related adverse events were observed during follow-up. Transient diarrhea occurred after nintedanib initiation and improved after dose reduction. This case suggests that biologically rational integration of JAK inhibition and anti-fibrotic therapy may be considered as a hypothesis-driven salvage approach in selected patients with progressive radiation pneumonitis with possible immune-mediated contribution after inadequate response to systemic corticosteroid therapy.

## Introduction

Radiation pneumonitis and immune checkpoint inhibitor-related pneumonitis are dose-limiting pulmonary toxicities in thoracic oncology, particularly when radiotherapy and ICIs are administered sequentially or concurrently (1). When ICI-related pneumonitis becomes corticosteroid-refractory, outcomes are poor; approximately 10% of corticosteroid-treated ICI-related pneumonitis cases require additional immunosuppression, and mortality after corticosteroid failure has been reported to reach 67% (2).

Corticosteroid resistance in this setting may be driven by persistent cytokine release and sustained JAK/STAT activation. JAK inhibition can attenuate IL-6/JAK/STAT3-mediated signaling and may enhance corticosteroid-induced immune-cell apoptosis in hyper-inflammatory contexts (3, 4). Elevated IL-6 and ferritin levels may help identify systemic immune activation in immune-related adverse events (3, 5). Because unresolved radiation- and ICI-associated inflammation may promote fibrotic remodeling (1), nintedanib, a VEGFR/PDGFR/FGFR-targeting multikinase inhibitor with established anti-fibrotic activity, may provide an adjunctive anti-remodeling strategy in selected refractory cases (6, 7).

Herein, we report a case of progressive radiation pneumonitis with possible immune-mediated contribution after inadequate response to moderate-dose systemic corticosteroid therapy, managed with a biologically rational, hypothesis-driven salvage strategy comprising JAK inhibition and adjunctive nintedanib.

## Case presentation

A 65-year-old man with recurrent esophageal cancer had previously undergone radical esophagectomy and multiple lines of systemic therapy, including pembrolizumab plus albumin-bound paclitaxel and nedaplatin. Pembrolizumab-based combination therapy was administered from November 2022 to October 18, 2023. The interval between the final pembrolizumab dose and symptom onset was approximately five months. After further disease progression, he received third-line thoracic radiotherapy from January to February 2024, with a total dose of 50 Gy delivered in 25 fractions.

One month after radiotherapy, the patient developed progressive cough, chest tightness, and dyspnea unresponsive to empirical antimicrobial therapy. HRCT showed left-predominant ground-glass opacities, focal consolidation, and traction bronchiectasis, largely within the radiation field but suggestive of overlapping radiation- and ICI-associated pneumonitis.

Laboratory testing showed a hyper-inflammatory profile, including elevated IL-6 (47.92 pg/mL), CRP (25.09 mg/L), neutrophil percentage, and ferritin (450.9 ng/mL). Sputum and blood cultures, serum 1,3-β-D-glucan, and galactomannan assays were negative, making opportunistic infection less likely. Bronchoscopy and bronchoalveolar lavage were not performed during the initial period of rapid clinical deterioration because of significant respiratory compromise and concern for procedure-related respiratory decompensation.

**Figure 1:**
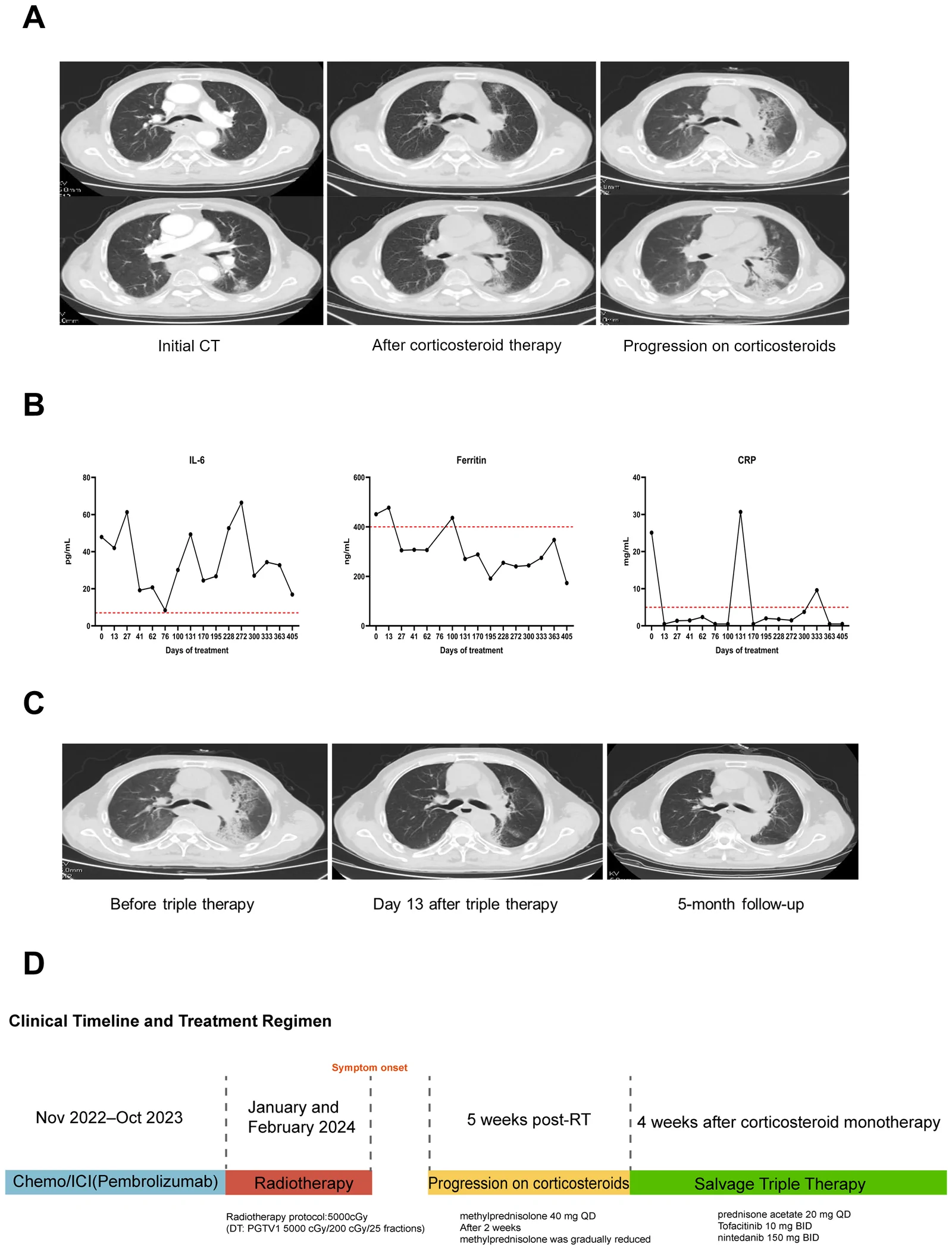
Clinical course, inflammatory biomarker dynamics, and radiographic response following hypothesis-driven salvage therapy. **(A)** Chest CT showing radiographic progression during corticosteroid monotherapy, with expanding ground-glass opacities and consolidations predominantly in the left lung. **(B)** Dynamic changes in interleukin-6, ferritin, and C-reactive protein after initiation of the hypothesis-driven salvage regimen. **(C)** Representative chest CT images showing radiographic response at three time points: before initiation of the hypothesis-driven salvage regimen, day 13 after treatment initiation, and at 5-month follow-up. **(D)** Clinical timeline summarizing pembrolizumab exposure, thoracic radiotherapy, symptom onset, progression despite corticosteroid therapy, initiation of hypothesis-driven salvage therapy, and subsequent clinical recovery.

Oral methylprednisolone 40 mg once daily plus empirical anti-infective prophylaxis was initiated. The patient’s body weight was 63 kg; therefore, the initial methylprednisolone dose corresponded to approximately 0.63 mg/kg/day, or approximately 0.79 mg/kg/day prednisone equivalent. This represented a moderate-dose systemic corticosteroid regimen. Four weeks later, symptoms worsened and repeat HRCT showed progressive consolidation with expanding ground-glass opacities, indicating progressive pneumonitis despite moderate-dose systemic corticosteroid therapy (**Figure 1A**).

At clinical deterioration, the patient had progressive dyspnea with a respiratory rate of 25 breaths/min and a decline in ECOG performance status to 3, markedly limiting daily activities. Based on the severity of symptoms and functional deterioration, the pneumonitis was clinically considered moderate-to-severe. However, retrospective CTCAE grading was limited by the absence of complete contemporaneous oxygenation data, including room-air SpO_2_, arterial blood gas analysis, and P/F ratio. The patient was clinically diagnosed with progressive radiation pneumonitis with possible immune-mediated contribution after inadequate response to moderate-dose systemic corticosteroid therapy. Because opportunistic infection could not be completely excluded and the patient was primarily managed in an outpatient setting, further corticosteroid escalation or pulse therapy was not pursued. After tapering corticosteroid therapy to prednisone acetate 20 mg once daily, a hypothesis-driven salvage regimen was initiated with tofacitinib 10 mg twice daily and nintedanib 150 mg twice daily.

Within two weeks of initiating the hypothesis-driven salvage regimen, the patient’s respiratory symptoms improved, with concurrent decreases in CRP, IL-6, and ferritin. A follow-up HRCT performed on day 13 after triple therapy showed marked regression of ground-glass opacities and consolidations (**Figure 1C**). Nintedanib was reduced to 100 mg twice daily because of transient uncomplicated diarrhea.

At 5 months, the patient remained largely asymptomatic. Prednisone acetate had been gradually tapered, and nintedanib was maintained at 100 mg twice daily after dose reduction. Tofacitinib was continued through the 5-month follow-up according to clinical response because IL-6 remained above the reference range. No clinically evident infectious complications, cytopenia, hepatic dysfunction, thrombotic events, or other serious adverse events were documented during follow-up. IL-6, ferritin, and CRP had decreased to 16.88 pg/mL, 173 ng/mL, and 0.5 mg/L, respectively (**Figure 1B**). HRCT showed near-complete resolution of inflammatory consolidations and stable residual fibrotic changes, while restaging scans showed radiographically stable esophageal malignancy (**Figure 1C**). The overall clinical course is summarized in **Figure 1D**.

## Discussion

Pulmonary toxicity after thoracic radiotherapy and ICI exposure represents a clinically relevant challenge. In a cohort of patients receiving thoracic radiotherapy combined with concurrent or sequential immune checkpoint inhibition, grade 2 or higher pneumonitis occurred in a minority of patients and no significant difference in adverse event rates was observed between concurrent and sequential treatment (8). These data suggest that combined thoracic radiotherapy and ICI treatment is often feasible, but clinically significant pneumonitis remains an important overlapping toxicity in selected patients. This case highlights a biologically rational, hypothesis-driven salvage strategy for progressive radiation pneumonitis with possible immune-mediated contribution after inadequate response to moderate-dose systemic corticosteroid therapy following thoracic radiotherapy and ICI exposure. Elevated IL-6 and ferritin levels supported a hyper-inflammatory phenotype and provided the rationale for moving from empirical corticosteroid-based therapy to targeted pathway modulation. Radiation pneumonitis and ICI-related pneumonitis differ in both pathobiology and clinical behavior. Radiation pneumonitis is generally considered a locoregional injury driven by radiation-induced epithelial and endothelial damage, inflammatory cytokine release, and subsequent profibrotic remodeling, and its radiographic distribution often corresponds to the irradiated field (1). In contrast, ICI-related pneumonitis reflects systemic immune dysregulation and T-cell–mediated inflammatory lung injury, with more heterogeneous radiographic patterns, including organizing pneumonia, nonspecific interstitial pneumonia, hypersensitivity pneumonitis, and diffuse alveolar damage (9). These mechanistic differences may influence treatment considerations: both entities are initially treated with corticosteroids, but corticosteroid-refractory ICI-related pneumonitis often prompts systemic immunosuppressive escalation (14–16), whereas radiation pneumonitis management may also require attention to radiation-associated fibrotic remodeling (1, 12, 13). In patients exposed to both thoracic radiotherapy and ICIs, these mechanisms may overlap, but the relative contribution of each component is often difficult to determine (1, 8).

In the setting of thoracic radiotherapy and prior ICI exposure, radiation-induced lung injury may be amplified by ICI-mediated immune activation and persistent cytokine release (1). Previous studies have associated corticosteroid resistance with excessive Th1 cytokine responses, elevated TNF-α and IFN-γ levels, and persistent activation of STAT3, NF-κB, and NLRP3 inflammasome pathways (10, 11). Sustained JAK/STAT activation may promote anti-apoptotic signaling and reduce corticosteroid-induced immune-cell apoptosis, whereas JAK inhibition may attenuate cytokine-mediated inflammatory signaling and potentially restore corticosteroid responsiveness in selected hyper-inflammatory contexts (4). Given the prior exposure to ICIs, overlap between radiation-induced injury and immune-mediated pneumonitis cannot be fully excluded, providing additional rationale for targeting JAK/STAT-mediated inflammatory signaling.

**Figure 2:**
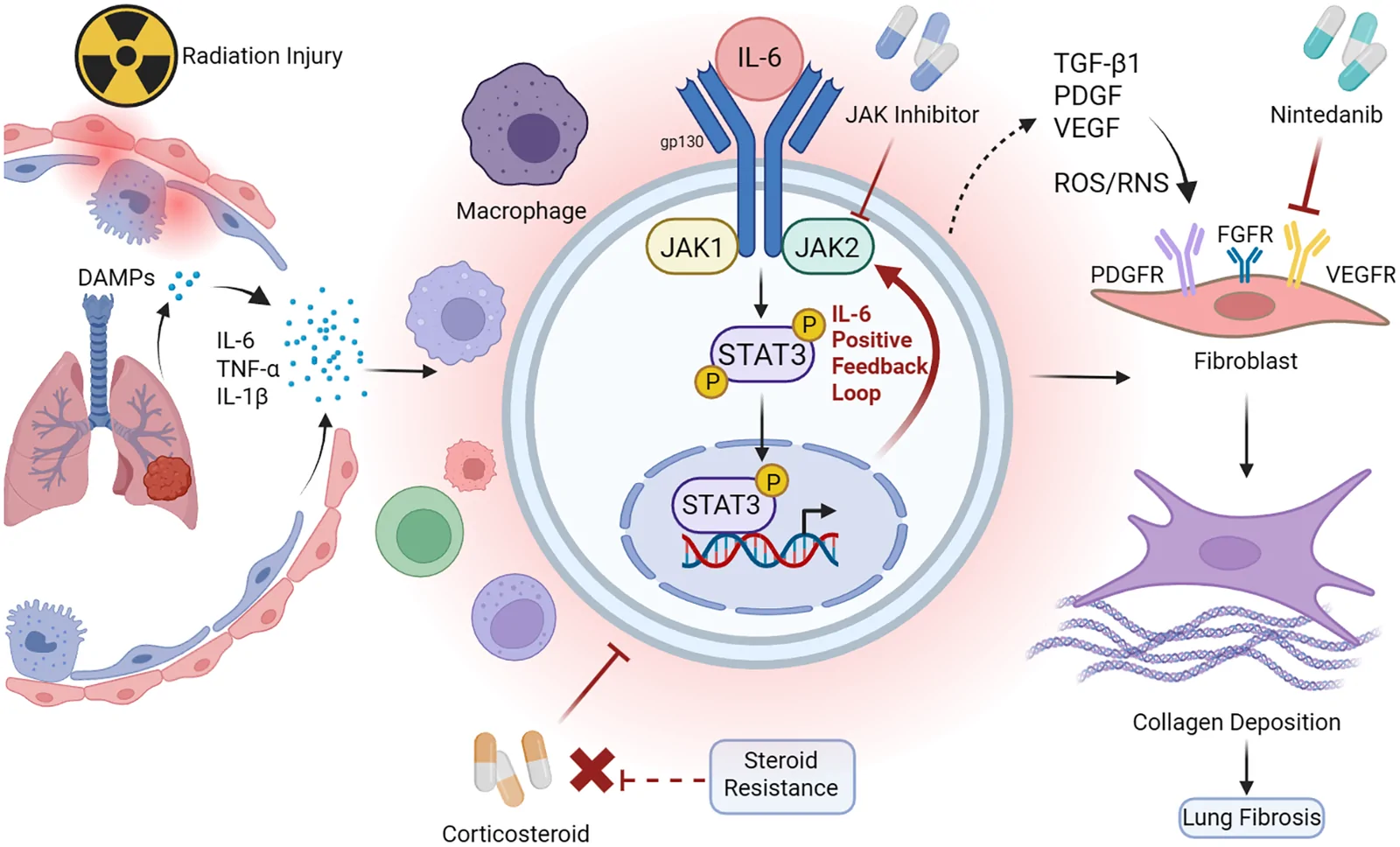
Proposed mechanistic model of biologically rational JAK inhibition plus nintedanib in progressive radiation pneumonitis with possible immune-mediated contribution. Radiation-induced lung injury triggers the release of damage-associated molecular patterns (DAMPs) and pro-inflammatory cytokines, including IL-6, TNF-α, and IL-1β. In patients with a hyper-inflammatory phenotype, persistent activation of the IL-6/JAK/STAT3 axis may contribute to a self-amplifying inflammatory circuit associated with reduced corticosteroid responsiveness. Activated STAT3 signaling may also interact with profibrotic pathways, promoting fibroblast activation and extracellular matrix deposition. JAK inhibitors may interrupt cytokine-mediated inflammatory signaling by reducing STAT3 activation, whereas nintedanib inhibits PDGFR, FGFR, and VEGFR signaling to limit fibroblast-to-myofibroblast transition and fibrotic remodeling. Collectively, this hypothesis-driven strategy provides a biological rationale for combined JAK inhibition and anti-fibrotic therapy in selected patients with hyper-inflammatory progressive radiation pneumonitis after inadequate response to systemic corticosteroid therapy. Created in BioRender. Yu, X. (2026) https://BioRender.com/4s7dwfl.

Unresolved radiation- and ICI-associated inflammation may promote fibrotic remodeling, providing a rationale for adjunctive nintedanib. Persistent IL-6/JAK/STAT3 activation may also interact with profibrotic pathways, including TGF-β- and PDGF-mediated fibroblast activation, thereby facilitating the transition from acute inflammatory injury to irreversible fibrosis. Therefore, early intervention targeting both cytokine-driven inflammation and profibrotic remodeling may be particularly relevant in hyper-inflammatory phenotypes. Through inhibition of VEGFR, PDGFR, and FGFR signaling, nintedanib may limit fibroblast activation, angiogenesis, and extracellular matrix deposition (1, 7). Clinical evidence for nintedanib in radiation pneumonitis remains limited and not yet definitive. In a phase 2 randomized, double-blind, placebo-controlled study evaluating nintedanib as prophylaxis against radiation pneumonitis in patients with unresectable non-small cell lung cancer undergoing chemoradiation, no clear significant benefit was demonstrated, partly limited by the small sample size (12). More recently, a randomized phase II placebo-controlled trial evaluated nintedanib added to a prednisone taper for grade 2 or higher radiation pneumonitis and suggested that the combination may reduce pulmonary exacerbations (13). These studies indicate that nintedanib has been clinically explored in the prevention and treatment of radiation pneumonitis and provide a rationale for targeting profibrotic signaling in radiation-associated lung injury. However, the available evidence remains limited, and nintedanib has not been established as a standard treatment for progressive radiation pneumonitis with possible immune-mediated contribution after inadequate response to systemic corticosteroid therapy. A proposed mechanistic model illustrating the interplay between cytokine-driven hyper-inflammatory state, JAK/STAT signaling, and fibrosis, as well as the rationale for combined JAK inhibition and anti-fibrotic therapy, is presented in **Figure 2**. Because clinically significant pneumonitis necessitated interruption of further antitumor therapy, nintedanib was considered a biologically reasonable adjunct in this oncologic context, given its antifibrotic and antiangiogenic properties. However, any potential antitumor contribution remains speculative, and tumor stability in this single case cannot be attributed to nintedanib.

Current guidelines recommend additional immunosuppression for corticosteroid-refractory ICI-related pneumonitis, but no standardized second-line regimen has been established (14, 15). Clinical data on corticosteroid-refractory or corticosteroid-resistant ICI-related pneumonitis remain limited. In a single-center retrospective series from an experienced thoracic oncology center (16), additional immune modulators such as TNF-α inhibitors and mycophenolate were associated with durable improvement in more than one-third of patients; however, 90-day all-cause mortality or hospice referral remained high, and severe infections were observed during intensified immunosuppression. These findings highlight both the potential value and limitations of broad immunosuppressive escalation. In contrast, direct evidence supporting JAK inhibition for progressive radiation pneumonitis with possible immune-mediated contribution remains scarce. Nevertheless, tofacitinib has been clinically explored in other corticosteroid-resistant irAEs, including ICI-associated myocarditis (17), supporting the broader rationale for JAK/STAT pathway modulation in selected hyper-inflammatory irAEs while underscoring the need for cautious extrapolation. Broad immunosuppression may increase infectious risk and does not directly address inflammation-associated fibrotic remodeling. Therefore, strategies targeting both cytokine-driven inflammation and downstream fibrotic responses may provide a more biologically rational approach for selected hyper-inflammatory cases. Building on emerging case-level evidence for anti-fibrotic therapy in immune-related pneumonitis (18), the present case suggests that combining JAK inhibition with nintedanib may represent a strategy to simultaneously target cytokine-mediated inflammation and early fibrotic remodeling.

A major limitation of this report is its single-case observational design. The working diagnosis of radiation/immune overlap pneumonitis was based on the temporal sequence of prior ICI exposure followed by thoracic radiotherapy, the hyper-inflammatory biomarker profile, and the clinical possibility that ICI exposure may have amplified radiation-associated lung injury. However, because the radiographic abnormalities largely corresponded to the irradiated region and no BAL or tissue-based immune profiling was available, radiation pneumonitis was considered the predominant component, while the contribution of ICI-related immune injury could not be established with certainty. Bronchoscopy and bronchoalveolar lavage were not performed during the initial period of rapid clinical deterioration because the patient was managed primarily in the outpatient setting and had significant respiratory compromise. The treating physicians considered that the procedure might increase the risk of respiratory decompensation, while the expected incremental diagnostic benefit was limited in the context of the available clinical, microbiological, and radiographic findings. Although biomarker decline and radiographic resolution were consistent with our mechanistic hypothesis, the absence of bronchoalveolar lavage transcriptomics, immune-cell phenotyping, or lung biopsy precluded direct confirmation of JAK/STAT pathway modulation in the lung microenvironment. Because tofacitinib and nintedanib were initiated simultaneously while corticosteroids were continued, the individual contribution of each agent could not be determined, and delayed corticosteroid efficacy or spontaneous improvement could not be fully excluded. In addition, baseline pulmonary function tests, including FVC and DLCO, serial oxygenation data, and specific fibrotic biomarkers such as KL-6 and SP-D were not available. LDH was not measured at the time of acute deterioration or initiation of the salvage regimen; therefore, it could not be used to evaluate the acute inflammatory response. These missing longitudinal physiological and biomarker data limited a more comprehensive assessment of inflammatory activity and fibrotic progression. Detailed dosimetric parameters such as lung V20 and mean lung dose were not available for this retrospective report, which limited precise correlation between CT abnormalities and dose distribution. Longer follow-up is needed to assess the durability of pulmonary recovery and oncologic safety. Ultimately, prospective studies are needed to further evaluate the safety, efficacy, and optimal patient selection for this hypothesis-driven strategy in progressive radiation pneumonitis with possible immune-mediated contribution after inadequate response to systemic corticosteroid therapy.

### Patient perspective

The patient experienced severe respiratory symptoms and significant anxiety during the period of rapid pneumonitis progression despite corticosteroid therapy. Following the initiation of the combined treatment strategy, his respiratory symptoms gradually improved, and he was able to resume his daily activities. The patient expressed satisfaction with the treatment outcome and agreed to share his clinical course to improve understanding of this challenging condition.

## Data Availability

The datasets presented in this article are not readily available because of ethical and privacy restrictions. Requests to access the datasets should be directed to the corresponding author.
